# The Manchester Crematorium

**Published:** 1892-08-27

**Authors:** 


### The Manchester Crematorium.

The Manchester Crematorium was used for the first time on Monday last, the 22nd inst. The body which was cremated was that of the late Mr. Thomas Morgan Brown, a gentleman of the age of eighty-four years, who had resided at Carlisle, whither he had returned from a forty years' residence in the United States. Information of the death, and of the desire for cremation, was received by Mr. J. Harvey Simpson, of 15, Princess Street, Manchester, the Secretary to the Company, on Saturday morning last, and the form of application for cremation, together with copies of two medical certificates, to be signed by two registered medical practitioners, were at once forwarded by them to the representatives of the deceased. The body was brought from Carlisle on Monday morning, under the charge of Messrs. Kendal, Milne, and Co., of Manchester, by whom it was taken to the Crematorium in a plain black hearse, attended by one coach, with an executor under Mr. Brown's will. On arrival at the Crematorium the coffin was placed on the catafalque at the far end of the hall, this being a raised slab of about ten feet in length, placed directly in front of the iron doors leading to the furnace. As soon as the coffin was placed in position, an electric bell was pushed by Mr. Henry Simon, the chairman of the Crematorium Board, and immediately the doors opened, the coffin glided into the receptacle, and was at once shut in. In about an hour all that remained of the body was a small quantity of calcined dust, which was placed in an urn and handed to Mr. Brown's executor. The whole arrangements of the cremation were marvellously simple, and, even during the disappearance of the coffin into the receptacle, no sight or even idea of consuming flames was conveyed to the spectators. Except for the ornamental laying out of the grounds the Crematorium is now completely finished, and ready for use at any time. The supporters of the cremation movement are to be congratulated upon the handsome appearance of the building, the completeness of the arrangements for cremation, and the simple and reverent manner in which they have arranged the same to be carried out.

